# A System of Operative Surgery

**Published:** 1852-03

**Authors:** 


*A System of Operative Surgery, based upon the Practice of Surgery in the United States; and comprising a Bibliographical Index and Historical Record of many of their operations, for a period of two hundred years.* By Henry H. Smith, M. D., Surgeon to the St. Joseph's Hospital, &c. Philadelphia: Lippincott, Grambo & Co. 1851.

It is a pleasure to meet with an American book like this; one which is really American, and not a mere transcript of British and European works. As the title indicates, it is based on the practice of surgery in the United States. It commences with a History of Surgery, and not the least interesting portion is a kind of Synopsis, or "Historical Record of some of the principal facts of interest connected with the origin and progress of Medicine and Surgery in the United States, arranged to facilitate reference." Then follows a "Bibliographical Index of American writers on subjects connected with Operative Surgery." The remainder of the work is divided into two parts; the first of which is devoted to the general duties of the surgeon and to elementary operations; and the second to the details of "Operations on the Head and Face." The mechanical execution of the volume is of the best kind, and it is illustrated by twenty-eight very nicely engraved steel plates. D.

